# Mucoepidermoid Carcinoma of the Right Bronchus in a Six‐Year‐Old Girl: A Rare Pediatric Case Report

**DOI:** 10.1155/crpe/5552173

**Published:** 2026-02-17

**Authors:** Daniela Kraljević, Ante Damjanović, Tamara Nikše, Ivan Pavić, Iva Mihatov Štefanović, Josip Pejić

**Affiliations:** ^1^ Department of Pulmonology and Allergology, Pediatric Clinic, University Clinical Hospital Mostar, Mostar, Bosnia and Herzegovina, skbm.ba; ^2^ Department of Pediatric Pulmonology, Allergology, Immunology and Rheumatology, Pediatric Clinic, Children’s Hospital Zagreb, Zagreb, Croatia, kdb.hr; ^3^ School of Medicine, University of Split, Split, Croatia, unist.hr; ^4^ Department of Endocrinology, Diabetes, Pulmonology and Allergology, Pediatric Clinic, CHC “Sestre Milosrdnice”, Zagreb, Croatia; ^5^ School of Dental Medicine, University of Zagreb, Zagreb, Croatia, unizg.hr; ^6^ Department for Thoracic Surgery, Surgery Clinic, Clinical Hospital Dubrava, Zagreb, Croatia, kbd.hr

**Keywords:** cancer, lungs, pediatric pulmonology

## Abstract

Primary lung neoplasms in children are uncommon, with a significant majority being metastatic. Among primary lung tumors, mucoepidermoid carcinoma (MEC) represents a rare entity, accounting for 0.1%–0.2% of cases. We present the case of a six‐year‐old girl who initially presented with right‐sided pneumonia and pleural effusion. Despite initial antibiotic therapy, she exhibited persistent respiratory symptoms, leading to further investigation. Chest CT revealed a spherical mass obstructing the right main bronchus, confirmed via bronchoscopy as a yellowish‐white formation. The pathological analysis indicated MEC. Surgical intervention involved a right thoracotomy with resection of the intermediate bronchus and inferior lobectomy. The patient recovered well postoperatively, with no tumor recurrence in follow‐up examinations. This case highlights the need for a high index of suspicion for MEC in children with recurrent respiratory symptoms localized to the same lung region. Thorough diagnostic evaluation, including bronchoscopy, is crucial for appropriate management, as surgical resection remains the primary treatment modality and can result in excellent outcomes. Early diagnosis and intervention are essential to improving prognosis in pediatric patients with lung neoplasms.

## 1. Introduction

Primary lung neoplasms in children are rare. A significantly larger number of lung neoplasms are secondary neoplasms, mostly metastases from some other primary tumor process [1]. Primary lung tumors in children, although rare, are mostly malignant (75% of cases). Carcinoids account for 40% of these tumors, bronchogenic carcinomas for 17% of cases, and pleuropulmonary blastomas for about 15% of cases [2]. Mucoepidermoid carcinoma (MEC) is the most common type of salivary gland carcinoma in the adult population. It can also be found in the bronchi and in the thyroid gland. It is not frequently found in the lungs, especially in children, where it constitutes approximately 0.1%–0.2% of all primary lung tumors [3]. It originates from glandular tissue identical to that of the salivary glands, which is located in the submucosa of the trachea and bronchi [3, 4]. We will present a rare case of MEC of the right bronchus in a six‐year‐old girl who was hospitalized due to right‐sided pneumonia and pleural effusion.

## 2. Case Presentation

**FIGURE 1:**
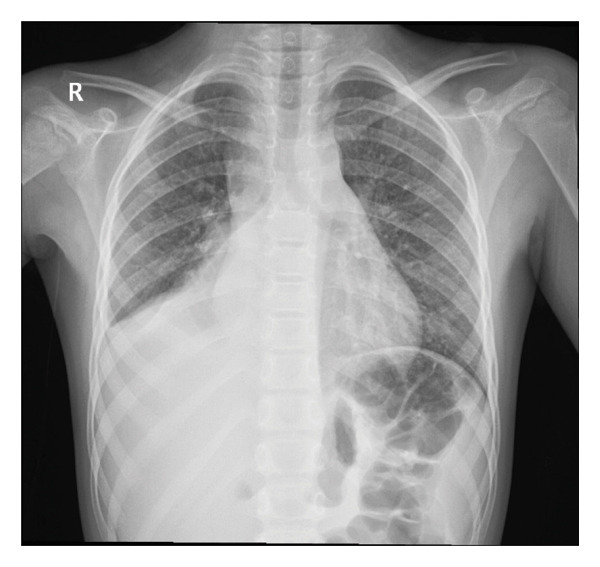
Posteroanterior (PA) chest radiograph shows increased homogeneous opacification of the right hemithorax with a lateralized pleural line and poor delineation of the right hemidiaphragm and right cardiac border, consistent with a pleural effusion.

A six‐year‐old patient was referred to our clinic due to an elevated body temperature, shivering, and vomiting. Laboratory diagnostics were performed at the local health center (CRP: 338 mg/L; WBC: 18.8 × 10^9/L [neutrophils 78%]), along with a chest X‐ray, which revealed right‐sided pneumonia with pleural effusion (Figure 1). The patient had previously experienced chickenpox 7 months before presentation and had two episodes of pneumonia since, which were treated on an outpatient basis.

The clinical examination of the patient revealed the following: Low‐grade fever (37.8°C), tachycardia (118/min), and reduced breath sounds on auscultation over the right lung, with no breath sounds heard at the base. Percussion on the right side of thorax revealed a dull sound. Additional radiological assessment (ultrasound of the lung base) confirmed the presence of pleural effusion.

Parenteral (ceftriaxone and clindamycin) and oral (azithromycin) antimicrobial therapy was prescribed, and a pediatric surgeon was consulted. There was no indication for pleural drainage.

The patient responded positively to the prescribed therapy, becoming afebrile on the fifth day of hospitalization. Follow‐up X‐rays showed partial regression of the previously described inflammatory changes, and a follow‐up ultrasound confirmed the regression of pleural effusion. The girl was discharged for home treatment with continued oral antimicrobial therapy (cefpodoxime), with a scheduled follow‐up appointment at the clinic.

Two months later, the patient was readmitted due to right‐sided pneumonia. On admission, she had a subfebrile temperature and appeared pale. There were reduced breath sounds over the right basal lung, and her abdomen was tender to palpation.

Laboratory diagnostics revealed a white blood cell count of 20.4 × 10^9^/L (neutrophils: 76%) and a CRP level of 293.7 mg/L. No pathogenic microorganisms were isolated in microbiological tests. A chest X‐ray showed persistent infiltrates on the right, with a suspicion of atelectasis of the middle lobe of the right lung. An ultrasound of the lung base revealed pleural effusion.

**FIGURE 2:**
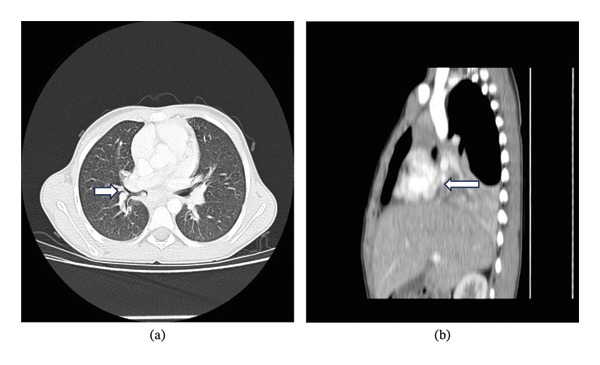
(a) Axial CT demonstrating heterogeneous content protruding into the right main bronchus, as well as the intermedius bronchus. (b) Sagittal CT demonstrating an atelectasis of the entire lower lung lobe on the right and the lateral segment of the middle lobe. CT: computerized tomography.

Empirical parenteral and oral antimicrobial therapy was prescribed. Due to the persistence of the infiltrates, a chest computerized tomography (CT) was performed. The CT revealed heterogeneous content protruding into the right main bronchus (Figure 2), as well as the intermedius bronchus, and was spherical in shape (possible mucous content, food aspiration). There were also smaller conglomerates of lymph nodes in the mediastinum and right hilum.

**FIGURE 3:**
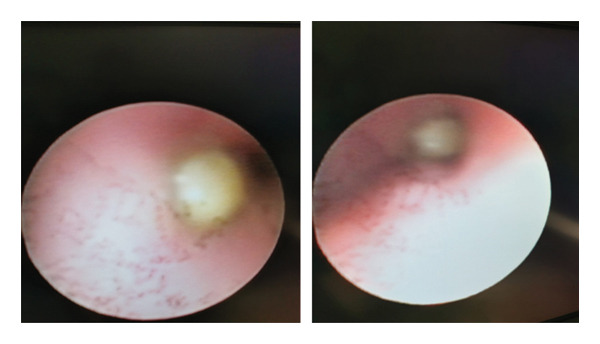
Flexible bronchoscopy: entrance to the right intermediate bronchus completely obstructed by a yellowish‐white formation.

Considering the findings of the chest CT, a flexible bronchoscopy was performed. The entrance to the right intermediate bronchus was completely obstructed by a yellowish‐white formation (possible mucous plug, foreign body). The formation protruded into the right main bronchus (Figure 3).

A recommendation was made for rigid bronchoscopy, which was performed under general anesthesia. A formation measuring 1.5 cm in length and up to 0.5 cm in thickness was extracted. A sample was sent for histopathological analysis.

Based on the histopathological analysis, the biopsy material revealed fragments of cylindrical mucinous epithelium, metaplastic squamous epithelium, and an abundant amorphous pink substance (possible necrotic tissue) surrounded by evident granulation tissue with areas of suppurative inflammation.

It was decided to send the sample for further analysis to the Institute of Pathology and Cytology at UHC Zagreb‐Rebro. A follow‐up flexible bronchoscopy was performed, which indicated that the entrance to the right intermediate bronchus remained obstructed by a compact yellowish‐white formation. The decision was made to postpone the rigid bronchoscopy until the pathological analysis results were available.

The patient was afebrile for several days, and follow‐up laboratory tests showed a decrease in inflammatory markers. However, there was another episode of fever, along with an increase in inflammatory markers. A combination antimicrobial therapy (clindamycin and meropenem) was prescribed, and the patient responded positively.

Since the initial biopsy material was insufficient for a correct analysis, it was decided to perform a rigid bronchoscopy and pathological material analysis at another medical center. The patient was referred to the “Sestre Milosrdnice” University Hospital Center in Zagreb.

Upon hospitalization at the new facility, the previously prescribed antimicrobial therapy was continued. A flexible bronchoscopy was performed, revealing a round, fibrous, and elastic formation in the right segmental bronchus, which filled the entire segmental bronchus (middle lobe on the right). Three samples were taken for analysis.

The pathological analysis results were consistent with MEC. The patient was transferred to the Children’s Hospital Zagreb for further treatment.

Under general anesthesia, a right‐sided thoracotomy was performed in the 5th intercostal space. The intermediate bronchus was resected, and an inferior lobectomy was performed. Macroscopically, the tumor did not extend to the resection margin (Sample number 1). Paratracheal lymph node dissection was carried out (Sample number 2). A chest tube was placed under negative pressure.

After the procedure, the patient was transferred to the intensive care unit. An ultrasound examination of the lungs confirmed complete consolidation of the upper lobe parenchyma and atelectasis on the right side. A flexible bronchoscopy was performed, and the right bronchus entrance was cleaned.

The patient’s condition gradually improved, and the chest tube was removed on the sixth postoperative day. The subsequent course of the patient’s stay was uneventful, and follow‐up laboratory results were normal. The margins of the pathological analysis preparations were clean, and there were no tumors in the lymph nodes; reactive changes were observed.

## 3. Discussion

Primary lung tumors in children are rare, and they are most often malignant in nature.

MEC of the bronchus accounts for about 10% of primary lung tumors in the pediatric population [3, 5].

MEC arises from the submucosal glands of the tracheobronchial tree, and because of its endobronchial location, it often presents with nonspecific symptoms such as persistent cough, recurring pneumonia, wheezing, or hemoptysis, leading to delayed diagnosis in children with recurrent respiratory symptoms [6, 7].

MEC primarily affects the large airways, leading to common symptoms such as cough, shortness of breath, fever, wheezing, chest pain, and hemoptysis [8].

Imaging, especially contrast‐enhanced chest CT, is crucial in identifying endobronchial masses and associated complications such as atelectasis or consolidation, guiding further endoscopic assessment and surgical planning in pediatric MEC cases [6, 7].

MECs can occur as both low‐ and high‐grade tumors. The low grade occurs in a younger population and the high grade in an older population. One‐third of patients are asymptomatic, and MECs rarely present as a peripheral pulmonary nodule. Less than 5% of low‐grade MECs spread to lymph nodes, and high‐grade MECs commonly metastasize distantly. Furthermore, low‐grade lesions are cystic, while high‐grade lesions are solid [9].

The classification of MEC into high grade or low grade depends on histological appearances, mitotic frequencies, cellular atypia, and necrocytosis [10].

Tumors are usually right‐sided with low histologic grade, although there are cases of high‐grade MEC documented among children [6].

Radiological diagnostics may not be sufficient to establish a definitive diagnosis, and endoscopic examination, such as flexible bronchoscopy, is often required.

A fiber optic bronchoscopy view of an MEC tumor is of a pedunculated, polypoidal, smooth, exophytic lesion that may resemble a carcinoid tumor [9].

Bronchoscopy with biopsy has been reported to be essential for diagnosis in pediatric MEC, confirming histology and ensuring correct tissue sampling prior to definitive surgical intervention [6, 7].

Chest X‐rays are typically normal in cases where MEC is located in the tracheal region, but most bronchial lesions are characterized by lobar infiltrates, atelectasis, or bronchiectasis due to partial or complete endobronchial obstruction [11].

Recurrent lung infections, especially in the same region, should raise suspicion of a possible underlying tumorous process.

MEC is often presented as an intraluminal mass leading to luminal occlusion [12].

In the differential diagnosis of endobronchial tumors in children, other possibilities include bronchial carcinoid tumors, inflammatory myofibroblastic tumors, foreign body aspiration, and endobronchial tuberculosis [8, 11, 12]. Bronchial carcinoids may have a similar endoscopic appearance but tend to have different immunohistochemical profiles and more common neuroendocrine features [8]. Inflammatory myofibroblastic tumors often present with similar symptoms and radiologic findings but have distinct pathological characteristics.

Metastasis to regional lymph nodes is rare in this type of tumor.

Due to the low metastatic potential of MEC, surgical treatment is usually the first option [8].

In some cases, bronchoscopy may be used for resection of small, hanging lesions in the lumen that do not infiltrate the bronchial wall [6].

The case discussed involved a right‐sided thoracotomy in the 5th intercostal space, resection of the intermediate bronchus, and an inferior lobectomy. Paratracheal lymph node dissection was performed.

Despite the rarity of this tumor, it is important to consider it in cases of persistent respiratory symptoms, especially when recurrent inflammatory processes occur in the same location.

Reported pediatric series show favorable long‐term outcomes following complete resection of low‐grade MEC with low rates of recurrence or metastasis, reinforcing the need for early surgical intervention and careful postoperative follow‐up [6, 7].

In this specific patient, the repeated episodes of pneumonia and persistent right‐sided infiltrates prompted comprehensive imaging and endoscopic evaluation, which ultimately led to the diagnosis of MEC. The case illustrates the importance of maintaining a high index of suspicion for endobronchial tumors in children with recurrent localized infections that do not respond fully to standard antimicrobial therapy.

Following complete surgical resection, the patient was closely monitored with serial imaging and flexible bronchoscopy. She remained in good health with no signs of recurrence or complications up to the time of writing this manuscript, consistent with favorable outcomes reported in other cases of low‐grade MEC following complete resection [5, 9]. Although no standardized schedule exists for follow‐up bronchoscopy in pediatric MEC, it is generally recommended to perform an initial postoperative bronchoscopy within the first few months to evaluate airway healing and to ensure there is no residual or recurrent lesion. Subsequent bronchoscopy and imaging may be individualized based on clinical symptoms, previous tumor grade, and the completeness of resection.

In low‐grade MEC with complete resection and negative margins, adjuvant radiation therapy or chemotherapy is typically not indicated. However, in cases of incomplete resection, high‐grade tumors, or metastatic disease, adjuvant therapies may be considered in consultation with a multidisciplinary pediatric oncology team. Close coordination between pediatric surgeons, pulmonologists, and oncologists is crucial to optimize outcomes and promptly address any recurrence or complications.

Overall, this patient’s course highlights the value of early recognition, the role of bronchoscopy for both diagnosis and follow‐up, and the generally favorable prognosis following complete resection of low‐grade MEC. Vigilant clinical follow‐up remains essential to ensure long‐term airway patency and to monitor for potential late complications.

The discussion emphasizes the importance of early recognition, accurate diagnosis, and appropriate surgical intervention in cases of MEC of the bronchus in pediatric patients.

## 4. Conclusion

MEC of the bronchus, though rare, should be considered in children with persistent or recurrent respiratory symptoms, especially those localized to the same lung region. This case underscores the importance of thorough diagnostic evaluation, including bronchoscopy, in cases where radiologic findings suggest airway obstruction. Surgical resection is a curative option, as demonstrated by the favorable outcome in this patient. Early diagnosis and treatment are keys to preventing long‐term complications and improving patient prognosis.

## Funding

No funding was received for this study.

## Disclosure

A preprint has previously been published in Authorea (Ante Damjanović, Daniela Kraljević, Tamara Nikše, et al. Mucoepidermoid carcinoma: A case report. Authorea. April 09, 2024) [13].

## Consent

Patient consent was obtained.

## Conflicts of Interest

The authors declare no conflicts of interest.

## Data Availability

The data that support the findings of this study are available from the corresponding author upon reasonable request.

## References

[1] Weldon C. B. and Shamberger R. C. , Mucoepidermoid Carcinoma in Childhood, Seminars in Pediatric Surgery. (2008) 17, no. 1, 17–29, 10.1053/j.sempedsurg.2007.10.004, 2-s2.0-37349079894. 18158138

[2] McCahon E. , Mucoepidermoid Carcinoma of the Bronchus: A Case Report, Paediatric Respiratory Reviews. (2006) 7, no. 3, 191–196, 10.1016/j.prrv.2006.05.002, 2-s2.0-33747778977. 16938641

[3] Qian X. , Sun Z. , Pan W. , Ye Q. , Tang J. , and Cao Z. , Childhood Bronchial Mucoepidermoid Tumors: A Case Report and Literature Review, Oncology Letters. (2013) 6, no. 5, 1409–1412, 10.3892/ol.2013.1529, 2-s2.0-84884475348. 24179533 PMC3813739

[4] Jayaprakash K. S. , Kishanprasad H. L. , Ismail M. , Raju M. , and Dasguptha A. , Mucoepidermoid Lung Carcinoma in a Child, Annals of Medical and Health Sciences Research. (2014) 4, no. 2, 276–278, 10.4103/2141-9248.129063. 24761253 PMC3991955

[5] Granata C. , Battistini E. , Toma P. et al., Mucoepidermoid Carcinoma of the Bronchus: A Case Report and Review of the Literature, Pediatric Pulmonology. (1997) 23, no. 3, 226–232. 9094733 10.1002/(sici)1099-0496(199703)23:3<226::aid-ppul10>3.0.co;2-9

[6] Wordui S. M. , Lakhan A. , Eze J. et al., Mucoepidermoid Carcinoma of the Bronchus in Two Children: Case Reports, Respiratory Medicine Case Reports. (2023) 43, 10.1016/j.rmcr.2023.101858. PMC10165254 37168990

[7] Huang Y. , Fu Y. , Sun J. , Xu B. , Wu L. , and Tang L.-F. , Pulmonary Mucoepidermoid Carcinoma in Children: Clinical Manifestations, Imaging and Treatment Outcomes—Case Series and Literature Review, Frontiers in Pediatrics. (2023) 11, 10.3389/fped.2023.1232185. PMC10522853 37772041

[8] Dinopoulos A. , Lagona E. , Stinios I. , Konstadinidou A. , and Kattamis C. , Mucoepidermoid Carcinoma of the Bronchus, Pediatric Hematology & Oncology. (2000) 17, no. 5, 401–408, 10.1080/08880010050034346, 2-s2.0-0033932385. 10914051

[9] Omesh T. , Gupta R. , Saqi A. , Burack J. , and Khaja M. , A Rare Case of Endobronchial Mucoepidermoid Carcinoma of the Lung Presenting as Non-Resolving Pneumonia, Respiratory Medicine Case Reports. (2018) 25, 154–157, 10.1016/j.rmcr.2018.08.014, 2-s2.0-85052472218. 30175037 PMC6115606

[10] Hu S. , Gong J. , Zhu X. , and Lu H. , Pulmonary Salivary Gland Tumor, Mucoepidermoid Carcinoma: A Literature Review, Journal of Oncology. (2022) 2022, 9742091–10, 10.1155/2022/9742091. 36385961 PMC9646301

[11] Kut A. , Karadag B. , Karakoc F. et al., Mucoepidermoid Carcinoma of the Bronchus: A Rare Entity in Childhood, Pediatrics International. (2005) 47, no. 2, 203–205, 10.1111/j.1442-200X.2005.02027. 15852520

[12] Kitada M. , Matsuda Y. , Sato K. et al., Mucoepidermoid Carcinoma of the Lung: A Case Report, Journal of Cardiothoracic Surgery. (2011) 6, no. 1, 10.1186/1749-8090-6-132, 2-s2.0-80053646524. PMC3207949 21985459

[13] Damjanović A. , Kraljević D. , Nikše T. , Pavić I. , Štefanović I. M. , and Pejić J. , Mucoepidermoid Carcinoma: A Case Report, Authorea. (2024) 10.22541/au.171266148.86228323/v1. PMC12910246 41710282

